# Comparative genetic characterization of Enteroaggregative *Escherichia coli* strains recovered from clinical and non-clinical settings

**DOI:** 10.1038/srep24321

**Published:** 2016-04-11

**Authors:** Rong Zhang, Dan-xia Gu, Yong-lu Huang, Edward Wai-Chi Chan, Gong-Xiang Chen, Sheng Chen

**Affiliations:** 1 Second Affiliated Hospital of Zhejiang University, Hangzhou, China; 2 Shenzhen Key lab for Food Biological Safety Control, Food Safety and Technology Research Center, Hong Kong PolyU Shen Zhen Research Institute, Shenzhen, P. R. China; 3 State Key Lab of Chirosciences, Department of Applied Biology and Chemical Technology, The Hong Kong Polytechnic University, Hung Hom, Kowloon, Hong Kong

## Abstract

The origin of pathogenic Enteroaggregative *Escherichia coli* (EAEC), a major causative agent of childhood diarrhea worldwide, remains ill-defined. The objective of this study was to determine the relative prevalence of EAEC in clinical and non-clinical sources and compare their genetic characteristics in order to identify strains that rarely and commonly cause human diarrhea. The virulence gene *astA* was commonly detectable in both clinical and non-clinical EAEC, while clinical isolates, but not the non-clinical strains, were consistently found to harbor other virulence factors such as *aap* (32%)*, aatA* (18%) and *aggR* (11%). MLST analysis revealed the extremely high diversity of EAEC ST types, which can be grouped into three categories including: (i) non-clinical EAEC that rarely cause human infections; (ii) virulent strains recoverable in diarrhea patients that are also commonly found in the non-clinical sources; (iii) organisms causing human infections but rarely recoverable in the non-clinical setting. In addition, the high resistance in these EAEC isolates in particular resistance to fluoroquinolones and cephalosporins raised a huge concern for clinical EAEC infection control. The data from this study suggests that EAEC strains were diversely distributed in non-clinical and clinical setting and some of the clinical isolates may originate from the non-clinical setting.

Diarrhea is one of the leading causes of child death in both developing and developed countries, causing an estimated 1.87 million deaths of children aged under 5 years globally each year^1^. Among the etiologic agents, diarrheagenic *Escherichia coli* (DEC) are most frequently implicated in cases of epidemic and endemic diarrheal diseases worldwide^2^. DEC strains are classified into six well-defined categories, including enteropathogenic *E. coli* (EPEC), verocytotoxigenic *E. coli* (VTEC), enterotoxigenic *E. coli* (ETEC), enteroaggregative *E. coli* (EAEC), enteroinvasive *E. coli* (EIEC) and diffusely adherent *E. coli* (DAEC)^2^.

Among the DEC strains, EAEC is increasingly recognized for its role in causing persistent diarrhea in children, traveler’s diarrhea and AIDS-associated diarrhea in both developing and industrialized countries^3^. In addition, it has been identified as the causative agent of several diarrhea outbreaks around the world^4^,^5^. Known to distribute widely in water^6^ and food^7^, and occasionally with animal feces^8^, diagnosis of EAEC has long been problematic. The gold standard method is the HEP-2 adherence test, in which EAEC strains exhibit a “stacked-brick” appearance in a characteristic aggregative adherence (AA) pattern^3^. However, the HEP-2 adherence test cannot distinguish between pathogenic and nonpathogenic EAEC strains, hence molecular biology assays based on detection of virulence determinants are now widely used to identify and characterize DEC isolates^9^,^10^,^11^,^12^.

The prevalence of antibiotic resistance in Enterobacteriaceae has increased globally in the past decade, presumably as a result of the frequent use of cephalosporins and fluoroquinolones in treatment of enteropathogenic bacterial infection, which is also commonly seen in hospitals in China^13^,^14^,^15^. Clinical EAEC isolates producing CTX-M-type extended spectrum β-lactamases (ESBLs) have been reported in Korea^16^ and Spain^17^. However, little data on the prevalence and genetic characteristics of EAEC isolates in China is available. In this study, we performed comparative characterization on both human clinical and environmental EAEC isolates to identify the possible origin of EAEC strains that commonly cause infections in China, and characterized the molecular basis of virulence and antimicrobial resistance in such organisms.

## Results

### Isolation of EAEC from clinical and environmental samples

**Figure 1:**
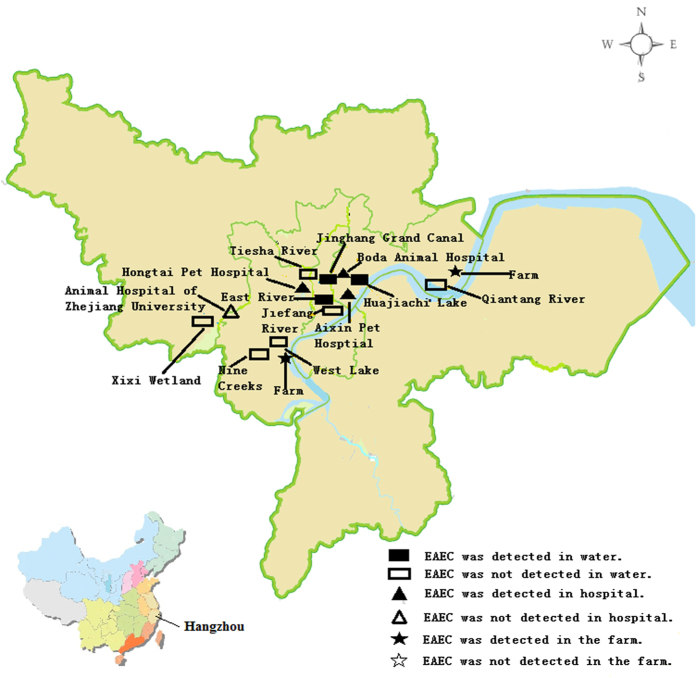
Distribution of sampling locations and sites where EAEC were recovered in Hangzhou, China. The figure was generated by obtaining the map of Hanzhou city from Zhejiang Administration of Surveying Mapping and Geoinformation (ZheS(2010)280) with the agency’s permission and further refined by Adobe Photoshop CS2 software.

A total of 790 non-duplicated *E. coli* isolates collected from both clinical and non-clinical settings were included in this study. Among them, 352 were of non-clinical origin including environmental water (n = 93), healthy animal feces (pigs, n = 68; companion animals, n = 85), and healthy human (n = 106; humans), the rest (438) were clinical isolated collected from diarrheal children <12 years old (n = 103), non-diarrhea inpatients (n = 66) and adults (n = 269). A total of 84 isolates were confirmed to be EAEC by HEP-2 adherence test, which depicted a characteristic aggregative adherence pattern for EAEC strains. Among these EAEC isolates, 33 were isolated from non-clinical settings with an isolation rate of 9%, whereas 51 (12%) were from diarrheal patients. The isolation rate of non-clinical samples was highest in swine specimens collected from pig farms (22%), followed by water (8%), companion animals (7%) and healthy human (5%). The prevalence of EAEC in pigs was significantly higher than all other sources (*P* ≦ 0.008). The two pig farms subjected to our survey were both positive for EAEC. Two out of the three animal hospitals were also positive for EAEC; in comparison, only three out of the nine water sampling sites were positive (Fig. 1). For clinical isolates, the isolation rate for diarrheal children and adult patients, at 13% (14/103) and 11% (30/269) respectively, was not significantly different from each other (*P* = 0.221). Surprisingly, EAEC isolation from non-diarrhea was about 11% (7/66), which was not lower than the isolation rate in diarrhea and significantly higher than health human (*P* ≦ 0.005).

### Prevalence of virulence genes in clinical and environmental EAEC

The most frequently detected virulence gene among the 84 EAEC isolates was *astA* (n = 75), higher than other reports, whereas *aggA* was not detectable in any of the isolates, a finding which was also reported in other studies suggesting *aggA* may not be a representative genetic marker for EAEC (Table 1)^18^,^19^. Clinical and non-clinical isolates exhibited a similarly high prevalence of *astA*, with a detection rate of 86% and 91% respectively. Yet a significant difference between the virulence gene profiles was observed between clinical EAEC isolates from diarrheal patients and those from non-diarrheal inpatients and non-clinical samples. The former were found to harbor other virulence factors such as *aap* (32%)*, aatA* (18%) and *aggR* (11%), while the latter contained exclusively astA gene (Table 1). The data suggested that other virulence factors such as *aap, aatA* and *aggR* could be the hallmarks of clinical EAEC causing human diarrhea. However, about 53% of the clinical EAEC from diarrheal patients contained only *astA.*

### Genetic relatedness of clinical and environmental EAEC

Upon observation of a discrepancy between virulence gene profiles of EAEC from clinical diarrheal patients and those from other sources, the degree of genetic relatedness between the EAEC isolates of different sources was determined by phylogenetic analysis. EAEC isolates from different sources were distributed amongst the phylogenetic groups (Table 2), with subgroups A and D being the most prevalent, containing 29 and 32 isolates, respectively. Subgroup B2, a common linkage with extra-intestinal infections, was similar both clinical and non-diarrheal isolates. No significant difference in the distribution of EAEC from different sources (Table 2).

All EAEC isolates were further typed by multilocus sequence typing to determine the linkage between clinical and non-clinical EAEC strains. A total of 60 different sequence types were identified among the 84 EAEC isolates. Most of the isolates belong to distinct ST type except for 12 STs that contained multiple isolates, with ST38 (7 isolates), ST10 (5 isolates), ST648 (4 isolates) and ST746 (3 isolates) being the most common ST types (Table 3). The majority of non-clinical EAEC isolates exhibited different STs of EAEC isolated from clinical isolates, supporting the idea that most of the non-clinical EAEC strains were different from clinical isolates. Importantly, our data indicated that 11 out of the 40 STs (28%) detectable in clinical isolates could also be seen among the non-clinical EAEC strains, which represented 38% of the 30 STs of the non-clinical organisms. Among them, the sequence type of ST38, which was found to cause diarrhea in both diarrheal children and out-patients, was also recoverable from water sources. Likewise, the sequence type of ST10, detectable in two out-patient cases, was observed in both water and pig farms, presumably due to the close proximity of these farms to the water sources (Table 3). ST155 and ST162 could be detected in both clinical diarrheal patients and companion animal suggesting the possible transmission route from pet. Other ST types such as ST48, ST117, ST131, ST744 and ST2496 could be detectable in both clinical diarrheal patients and environmental samples such as animal and water. Among clinical isolates, ST117 and ST131 could be detected in both diarrheal and non-diarrheal patients. ST131 was not detected in healthy human and animal samples, while it was seen in clinical sample and water sources suggesting the possible transmission of this clinical important ST type. Interesting, EAEC from healthy human did not show any common ST types from other sources (Table 3). These data indicated that transmission of EAEC from companion animals, farm animals and water sources to human patients was possible, while EAEC from healthy human was less likely to cause diarrhea. Further study will be needed to understand the different genetic features that constituted the difference. PFGE characterization was also performed for isolates sharing similar ST types. All these isolates showed <50% similarity, indicating that they were not epidemiologically related (data not shown).

In addition to the common ST types found from clinical and non-clinical EAEC, some ST types such as ST648 was consistently detectable in diarrheal out-patients and children, but not among environmental strains, suggesting that organisms belonging to this ST may not be environmental origin or reside in an environmental source not covered in this study (Table 3). Among the non-clinical EAEC strains, several ST types of EAEC recovered from healthy individuals, such as ST52, ST717 and ST767, were not detectable in diarrhea patients, suggesting that these ST types of EAEC may not be pathogenic to human. In addition, isolates with the novel sequence types ST4215, ST4216, ST4217, and ST4221 were collected from diarrheal patients, whereas an isolate with novel sequence type ST4214 was isolated from swine feces. These data suggested the high diversity of EAEC and different sources contained unique ST types of EAEC.

**Figure 2:**
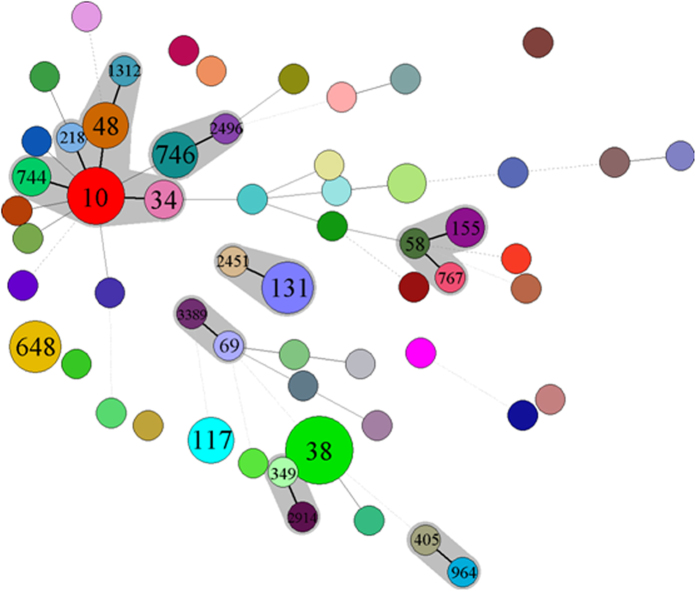
Minimal spanning tree based on multilocus sequence typing of EAEC isolates. Colored circles represent different sequence types. Black connecting lines indicate single-locus variants; gray connecting lines indicate double-locus variants; dashed connecting lines indicate strains with ≥3 differing loci; and shadowing indicates that >2 sequence types belong to one clonal complex.

To further look into the linkage between different ST types, minimal spanning tree was created to show the relationship between these 60 ST types detected in this study as shown in Fig 2. One big cluster was found including ST10, ST34, ST48, ST218 and ST744, which were clearly seen to be distributed in both clinical EAEC and EAEC strains from animal and water sources. ST767 EAEC from health human and ST58 from swine samples could further linked to clinical ST155. ST746 from non-clinical EAEC could be further linked to clinical ST2496. ST131 could further linked to ST2451, while ST38 and ST117, which contained a lot of strains, were relatively independent. These data further proved the linkage between clinical and non-clinical EAEC in particular swine and water EAEC isolates.

### Antimicrobial susceptibility profiles and dissemination of CTX-M type β-lactamases in clinical and non-clinical EAEC strains

The rate of resistance to fluoroquinolones in clinical EAEC isolates was lower than that of environmental EAEC strains, whereas resistance to cephalosporins was at a similar rate (>50%) for both groups (Table 4). Resistance to carbapenems and amikacin were the lowest among all the antibiotics tested. Since mechanisms of cephalosporin resistance were mainly mediated by the production of ESBLs in *E. coli*, the prevalence of different ESBL genes in EAEC was investigated. Cephalosporin resistant EAEC strains were found to harbor different variants of CTX-M genes, yet none of the isolates was positive for plasmid-mediated *AmpC* genes. The distribution of CTX-M gene types in both clinical and non-clinical EAEC was similar, with *bla*_CTX-M-14_ being the most prevalent type, and detectable in all samples types (Table 4). PMQR genes were also screened in these isolates, with results indicating that the *oqxAB* element was prevalent amongst EAEC isolates, and most frequently isolated from swine (6/15). Importantly, three PMQR determinants (*qnrS2*, *oqxAB*, and *aac*(*6′*)*-Ib-cr*) were detected simultaneously in two EAEC strains recovered from swine.

## Discussion

The current study revealed the existence of three distinct categories of EAEC strains: (i) non-clinical EAEC that rarely cause human infections; (ii) virulent strains recoverable in diarrhea patients that are also commonly found in the environment, such as ST38, ST10 and ST131 strains; (iii) organisms causing human infections but rarely recoverable in the environment. This phenomenon may suggest an uneven distribution of genetic traits/virulence genes among EAEC strains but govern the clinical infections of EAEC.

Organisms belonging to the subgroups A and B1 are generally regarded as commensal strains, whereas subgroup B2 is most frequently associated with extraintestinal infections^20^,^21^,^22^,^30^. However, we found that there was no dramatic difference of the distribution of phylogenetic groups of EAEC between clinical and non-clinical EAEC strains. The interesting finding from this study is to see the similar proportion of B2 group of EAEC from both clinical and non-clinical isolates. Nevertheless, the concept of uneven virulence among clinical and non-clinical EAEC strains is consistent with our observation of a discrepancy between the distribution patterns of known virulence genes among clinical and environmental isolates. Among the environmental EAEC strains tested, swine feces can be regarded as the most prominent source of EAEC, with all 15 isolates recoverable from this specimen type containing the *astA* gene, which encodes the EAST-1 toxin. This finding is in agreement with numerous other studies which showed that isolates recovered from farm animals frequently carried this virulence gene^12^,^23^. As *E. coli* isolates carrying *astA* alone were reported to cause diarrhea in Japan^24^, our finding that this element is extensively distributed amongst EAEC strains recovered from all environmental sources indicates that all EAEC strains can theoretically cause diarrhea, but the severity of the disease may depend on the gene copy number, expression level, and degree of co-existence with other virulence elements. It should be noted that virulence genes were identified in organisms recovered from diarrheal patients at a significantly higher frequency when compared to those harbored by healthy individuals. Fifteen isolates with more than one virulence gene were isolated from human patients, with the combination of *aap* and *astA* being most prevalent. Further investigation is required to identify factors that determine the functional relationship between *astA* and the degree of pathogenicity of EAEC.

Previous studies have supported the idea that highly resistant bacteria may harbor less virulent factors^25^,^26^, however the results of this study suggested that this is not necessarily true. We even found that an isolate with the *aap-aatA-aggR-pic* virulence gene profile produced the CTX-M-9-type ESBLs and TEM-1 enzyme. Therefore, no direct relationship between virulence genes and antimicrobial susceptibility was indicated. The prevalence of ESBLs-producing EAEC isolates in the current study (50%) was identical to that among *E. coli* isolates from various sources in China (~50%)^27^. In addition, ESBLs genes were more common amongst EAEC isolates from water, pigs, and healthy humans than in other *E. coli* isolates from the same sources (28.6% vs. 12.5%, 57.1% vs. 32.0%, 20% vs. 17.5%, respectively). ST131 is commonly associated with ESBLs production^28^. Since the first report of a CTX-M-15-producing EAEC isolate in the United Arab Emirates in 2006^29^, several other pathogens carrying CTX-M-15-type ESBLs have been identified^30^. In this study, one EAEC isolate carrying *bla*_CTX-M-15_ belonged to ST38, while six other ST38 isolates contained CTX-M-14-type ESBLs. It should be noted that this is the first study to identify ESBLs-producing EAEC isolates in China, which should be monitored closely.

EAEC isolates of the same sequence type tend to be isolated from locations that are in close proximity to each other. For example, ST746 isolates were recovered from swine samples in a farm near the Jinghang Grand Canal, as well as from water specimens collected from the Jinghang Grand Canal itself. This phenomenon may be explained by the theory that microbes from pig manure were dispersed into nearby rivers which subsequently caused contamination of a much larger area. The expansion of the resistance gene reservoir in the environment is likely caused by antibiotic use in both humans and animals^31^. China is the largest antibiotic producer and consumer in the world. Such high usage of antimicrobial drugs has exerted a strong pressure for selection of resistant microbes. We previously reported high prevalence of *qnr* and *aac*(*6′*)*-Ib-cr* in both water-borne bacteria and clinical isolates^32^. In the current study, over 60% of the EAEC isolates from swine and animals were resistant to both ciprofloxacin and levofloxacin, a level higher than that of diarrheal patients. Accordingly, *oqxAB* was most frequently identified in EAEC isolates from swine, presumably as a result of the extensive use of quinoxalines and quinolones in farm animals in China^33^.

In conclusion, we have compared the prevalence and molecular characteristics of clinical and environmental EAEC strains collected in Hangzhou, China. The similarity and uniqueness in the distribution patterns of sequence types among the test strains indicated that dynamic transmission of specific virulent strains between humans, animals, and the environment is possible. High antimicrobial resistance and ESBL prevalence was observed in both environmental and clinical EAEC isolates. The high detection rate of ESBL determinants and PMQR genes in EAEC highlights a need to impose stricter infection control measures to prevent further dissemination of the multiple drug resistant and highly virulent EAEC strains. More research efforts are needed to understand the antibiotic resistance status of this type of important, but less studied pathogens. The study warrants the prudent use of antibiotics in both animal and human.

## Material and Methods

### Clinical and environmental *E. coli* strain isolation

Non-clinical EAEC isolates were collected from different sources, including water, healthy humans and animals in Hangzhou, China. Water samples were collected from nine distinct water sources in Hangzhou (West Lake, Qiantang River, Jinghang Grand Canal, Xixi Wetland, Jiefang River, Huajiachi Lake, Nine Creeks, Tiesha River, and East River; Fig. 1) in November 2013. Two to three representative sites at each water source were selected for water sampling, and 5–10 one-liter samples were collected at each site. Bacteria in water samples were concentrated by vacuum filtration using a filter membrane (<0.2 μm). The membrane was then washed and suspended in 10 ml sterile 0.45% saline solution, and 200 μl of the suspension was inoculated onto MacConkey plates (Oxoid, Basingstoke, UK). A maximum of 6 *E. coli* like colonies from each plate were isolated for further identification. Animal fecal samples were collected from companion animals and pigs in four animal hospitals and two pig farms during the period of June to December 2013 (Fig. 1). Human fecal samples were collected from healthy individuals who have undergone routine physical examination during the similar period of time in 2013. Human and animal fecal samples were collected with sterile swabs and inoculated onto MacConkey, plates within 2 hours. A maximum of 6 *E. coli* like colonies from each plate were isolated for further identification. Clinical *E. coli* isolates were collected from fecal samples of outpatients with acute diarrheal diseases and non-diarrhea inpatients in the Second Affiliated Hospital of Zhejiang University from June to December 2013. Fecal samples from diarrheal patients under 12 years old were collected at the Children’s Hospital of Zhejiang University. All these samples were subjected to *E. coli* isolation as described above. The isolation sites have been shown in Fig. 1, which was generated using Adobe Photoshop CS2 software. All human samples were collected in accordance with relevant guidelines and regulations of the Second Affiliated Hospital of Zhejiang University, Hangzhou, China. All experimental protocols were approved by the Second Affiliated Hospital of Zhejiang University, Hangzhou, China. Informed consent has been obtained from all human subjects.

*E. coli* like colonies derived from each sample were identified using a VITEK 2 Compact system (bioMérieux, Hazelwood, MO, USA), and by matrix-assisted laser desorption ionization-time of flight mass spectrometry (BrukerDaltonik GmbH, Germany)^34^. When two or more strains of *E. coli* were isolated from the same sample, colony morphology and antimicrobial susceptibility testing were performed to remove duplicate strains exhibiting identical phenotypic characteristics.

### EAEC confirmation

All *E. coli* isolates from clinical and non-clinical samples were screened to identify EAEC strains by amplification of known EAEC specific genetic markers. *E. coli* strains were first subjected to PCR assay targeting to *aatA* (coding for a dispersin transporter protein) using primers (F-CAGACTCTGGCRAAAGACTGTATCAT/R- CAGCTAATAATGTATAGAAATCCGCTGT with product of 642bp)^35^, a genetic marker for 75 kb pAA plasmid^10^. *AatA*-negative *E. coli* strains will be further tested for the presence of other pAA-encoded virulence loci including *aggR* and *aap.* In addition, other virulence genes including *astA* (encoding heat-stable toxin EAST-1), *pic* (coding for a serine protease precursor), *aggA* (encoding Fimbria AAF/I) and *aafA* (encoding Fimbria AAF/II) were also screened by PCR assay as previously described^11^,^12^,^36^. PCR products were sequenced by Sangon Biotech Co. (Shanghai, China), and were compared with existing sequences in the GenBank database (http://blast.ncbi.nlm.nih.gov/Blast.cgi). *E. coli* isolates containing at least one of these virulence genes were further confirmed to be EAEC by the HEP-2 adherence test as previously reported^37^. *E. coli* isolates were confirmed to be EAEC and included in this study when aggregative adherence pattern was observed on HEp-2 cells.

### Molecular typing

A set of housekeeping genes including *adk* (encoding adenylate kinase), *fumC* (fumaratehydratase), *gyrB* (DNA gyrase), *icd* (isocitrate dehydrogenase), *mdh* (malate dehydrogenase), *purA* (adenylosuccinatesynthetase), and *recA* (ATP/GTP binding motif) was chosen as targets for multilocus sequence typing (MLST) which was performed as previously reported^38^. Sequence types (ST) were compared with those in the *E. coli* MLST database (http://mlst.ucc.ie/mlst/dbs/Ecoli). Phylogenetic grouping was performed by a multiplex PCR assay of the *chuA*, *yjaA*, and TspE4. C2 elements as previously reported^39^. Pulsed-field gel electrophoresis (PFGE) was performed using a Rotaphor System 6.0 instrument (WhatmanBiometra, Goettingen, Germany) according to a previous report^40^.

### Antimicrobial susceptibility testing

Antimicrobial susceptibility testing of EAEC was performed using the standard disk diffusion method on Mueller-Hinton agar (Oxoid). Susceptibility to ciprofloxacin, levofloxacin, cefotaxime, ceftazidime, cefuroxime, gentamycin, amikacin, ampicillin,imipenem, and meropenem disks was determined according to the manufacturer’s recommendations (Oxoid), and in accordance with the Clinical and Laboratory Standards Institute (CLSI) guidelines^41^. All experimental protocols were approved by CLSI. *E. coli* strain ATCC 25922 was used as a quality control. CT/CTL and TZ/TZL Etest strips (AB Biodisk, Solna, Sweden) were used for phenotypic detection of ESBL production among EAEC isolates.

### Detection of drug resistance determinants

Primers specific to the antibiotic resistance genes encoding the TEM, SHV, CTX-M-1, CTX-M-2, CTX-M-8, CTX-M-9 β-lactamases, as well as the plasmid-mediated AmpC enzyme, were used to determine cephalosporin-resistant genotypes as previously described^42^,^43^. Existence of plasmid-mediated quinolone resistance (PMQR) genes *qnrA*, *qnrB*, *qnrC*, *qnrD*, *qnrS, aac(6′)-Ib*, *qepA*, *oqxA*, and *oqxB* was determined as reported previously^44^,^45^,^46^. Carbapenemase genes (*bla*_KPC_, *bla*_IMP_, *bla*_VIM_, and *bla*_NDM_) were also amplified, as previously described^47^. Amplicons were sequenced as described above and compared with corresponding sequences in the GenBank database.

### Statistical analyses

The Chi-square test and Fisher’s exact t-test were used to determine whether differences in EAEC prevalence and ESBLs detection rates were statistically significant, using SPSS for Windows (version 19.0; SPSS Inc., Chicago, IL, USA). MLST profiles were analyzed by Bionumerics (Applied Maths, Sint-Martens-Latem, Belgium).

## Additional Information

**How to cite this article**: Zhang, R. *et al.* Comparative genetic characterization of Enteroaggregative *Escherichia coli* strains recovered from clinical and non-clinical settings. *Sci. Rep.* **6**, 24321; doi: 10.1038/srep24321 (2016).

## Figures and Tables

**Table 1:** Detection of virulence genes in 84 EAEC isolates recovered from different sources.

Sampling Sources (No. of isolates)	No. of EAEC isolates carrying virulence gene (%)						
	*astA*	*aggR*	*pic*	*aatA*	*aafA*	*Aap*	*aggA*
Clinical EAEC isolates (n = 51)	45 (88)	5 (10)	4 (8)	8 (16)	1 (2)	14 (27)	0 (0)
Diarrheal outpatients (n = 30)	26 (87)	4 (13)	1 (3)	7 (23)	1 (3)	10 (33)	0 (0)
Diarrheal children (n = 14)	12 (86)	1 (7)	3 (21)	1 (7)	0 (0)	4 (29)	0 (0)
Non diarrheal inpatients (n = 7)	7 (100)	0 (0)	0 (0)	0 (0)	0 (0)	0 (0)	0 (0)
Non-clinical EAEC isolates (n = 33)	30 (91)	0 (0)	3 (9)	0 (0)	0 (0)	0 (0)	0 (0)
Healthy human (n = 5)	3 (60)	0 (0)	2 (40)	0 (0)	0 (0)	0 (0)	0 (0)
Companion animals (n = 6)	5 (83)	0 (0)	1 (17)	0 (0)	0 (0)	0 (0)	0 (0)
Swine (n = 15)	15 (100)	0 (0)	0 (0)	0 (0)	0 (0)	0 (0)	0 (0)
Water (n = 7)	7 (100)	0 (0)	0 (0)	0 (0)	0 (0)	0 (0)	0 (0)
Total (n = 84)	75 (89)	5 (6)	7 (8)	8 (10)	1 (1)	14 (17)	0 (0)

**Table 2:** Distribution of phylogenetic subgroups among EAEC isolates from different sampling sources.

Sampling sources	No. of isolates (%)			
	A	B1	B2	D
Clinical EAEC isolates (n = 44)	21 (48)	6 (14)	7 (16)	17 (39)
Diarrheal outpatients (n = 30)	13 (43)	5 (17)	3 (10)	9 (30)
Diarrheal children (n = 14)	5 (36)	0 (0)	2 (14)	7 (50)
Non-diarrheal inpatients (n = 7)	3 (43)	1 (14)	2 (29)	1 (14)
Non-clinical EAEC isolates (n = 33)	8 (24)	6 (18)	4 (12)	15 (46)
Healthy human (n = 5)	2 (40)	1 (20)	1 (20)	1 (20)
Companion animals (n = 6)	1 (17)	0 (0)	1 (17)	4 (67)
Swine (n = 15)	3 (20)	4 (27)	2 (13)	6 (40)
Water (n = 7)	2 (29)	1 (14)	0 (0)	4 (57)
Total (n = 84)	29 (35)	12 (14)	11 (13)	32 (38)

**Table 3:** Distribution of sequence types among 51 clinical and 33 non-clinical/environmental EAEC isolates.

Sampling sources (no. of isolates)	Sequence Types (no. of isolates)
Clinical EAEC isolates (n = 44)	Total of 41 STs
Diarrheal outpatients (n = 30)	**ST10** (2); ST31 (1); **ST34** (1); **ST38** (1); ST40 (1); **ST48** (1); ST101 (1); **ST131** (1); **ST155** (1); ST201 (1); ST216 (1); ST349 (1); ST394 (1); ST414 (1); ST648 (3); ST964 (1); ST1193 (1); ST1312 (1); ST2141 (1); ST2451 (1); **ST2496** (1); ST2758 (1); ST2914 (1); ST3389 (1); ST4215 (1); ST4216 (1); ST4217 (1)
Diarrheal children (n = 14)	**ST38** (5); ST73 (1); **ST117** (1); **ST162** (1); ST405 (1); ST485 (1); ST501 (1); ST641 (1); ST648 (1); ST4221 (1)
Non-diarrheal inpatients (n = 7)	ST69 (1); **ST117** (1); **ST131** (2); ST206 (1); ST453 (1); **ST744** (1)
Non-clinical EAEC isolates (n = 33)	Total of 30 STs
Companion animals (n = 6)	ST12 (1); **ST155** (1); ST156 (1); **ST162** (1); ST359 (1); ST2248 (1)
Healthy human (n = 5)	ST52 (1); ST717 (1); ST767 (1); ST998 (1); ST2253 (1)
Swine (n = 15)	ST7 (1); **ST10** (1); **ST34** (1); **ST48** (2); ST58 (1); ST71 (1); ST88 (1); **ST117** (1); ST617 (1); **ST744** (1); ST746 (2); ST1112 (1); ST4214 (1)
Water (n = 7)	**ST10** (2); **ST38** (1); ST115 (1); **ST131** (1); ST746 (1); **ST2496** (1)

Sequence types recoverable in both clinical and non-clinical strains are shown in bold fond. For example, ST10 was recovered in both clinical (diarrheal patients) and non-clinical (swine and water) sources. Sequence types present in two or more sub-groups of the clinical or non-clinical strains are underlined. For example, ST38 was found in diarrheal outpatients and diarrheal children from clinical sources; ST746 was identified from swine and water of non-clinical sources.

**Table 4:** Antimicrobial susceptibility of 84 EAEC strains isolated of different origins.

Sampling sources	Resistance rate (%)										CTX-M variants (No. of isolates)
	CIP	LEV	CTX	CAZ	CXM	GEN	AMK	AMP	IPM	MEM	
Clinical EAEC isolates (51)	30	30	50	14	55	27	5	84	0	0	M-14, 15, 24, 55, 65
Diarrheal outpatients (n = 30)	30	30	40	10	47	17	3	83	0	0	M-14 (10), M-55 (2)
Diarrheal children (n = 14)	29	29	71	21	71	50	7	85	0	0	M-14 (5), M-55 (1), M-65 (1), M-24 (1), M-15 (1)
Non-diarrheal inpatients (n = 7)	43	43	57	43	57	0	0	86	29	29	M-14 (1), M-55 (1), M-3 (1), NDM-1(2)
Non-clinical EAEC isolates (33)	53	53	53	9	53	43	7	87	0	0	M-14, 24, 55, 64, 65
Healthy human (n = 5)	20	20	20	0	20	20	0	40	0	0	M-14 (1), M-55 (1)
Companion animals (n = 6)	67	67	67	50	50	50	0	67	0	0	M-24 (1), M-64(1), M-65 (1)
Swine (n = 15)	60	60	60	0	60	27	7	93	0	0	M-14(6), M-24 (1), M-64(1), M-65 (1)
Water (n = 7)	29	29	29	29	43	57	14	86	0	0	M-14 (1)

CIP, ciprofloxacin; LEV, levofloxacin; CTX, cefotaxime; CAZ, ceftazidime; CXM, cefuroxime; GEN, gentamycin; AMK, amikacin; AMP, ampicillin; IPM, imipenem; MEM, meropenem.
